# Plant growth promoting rhizobacterium *Stenotrophomonas maltophilia* BJ01 augments endurance against N_2_ starvation by modulating physiology and biochemical activities of *Arachis hypogea*

**DOI:** 10.1371/journal.pone.0222405

**Published:** 2019-09-12

**Authors:** Ankita Alexander, Vijay Kumar Singh, Avinash Mishra, Bhavanath Jha

**Affiliations:** 1 Biotechnology and Phycology Division, CSIR- Central Salt and Marine Chemicals Research Institute, G. B. Marg, Bhavnagar, Gujarat, India; 2 Academy of Scientific and Innovative Research (AcSIR), CSIR, Ghaziabad, India; ICAR-Indian Institute of Agricultural Biotechnology, INDIA

## Abstract

*Arachis hypogea* (Peanut) is one of the most important crops, and it is harvested and used for food and oil production. Being a legume crop, the fixation of atmospheric nitrogen is achieved through symbiotic association. Nitrogen deficiency is one of the major constrains for loss of crop productivity. The bacterium *Stenotrophomonas maltophilia* is known for interactions with plants. In this study, characteristics that promote plant growth were explored for their ability to enhance the growth of peanut plants under N_2_ deficit condition. In the presence of *S*. *maltophilia*, it was observed that fatty acid composition of peanut plants was influenced and increased contents of omega-7 monounsaturated fatty acid and omega-6 fatty acid (γ-Linolenic acid) were detected. Plant growth was increased in plants co-cultivated with PGPR (Plant Growth Promoting Rhizobacteria) under normal and stress (nitrogen deficient) condition. Electrolyte leakage, lipid peroxidation, and H_2_O_2_ content reduced in plants, co-cultivated with PGPR under normal (grown in a media supplemented with N_2_ source; C+) or stress (nitrogen deficient N+) conditions compared to the corresponding control plants (i.e. not co-cultivated with PGPR; C–or N–). The growth hormone auxin, osmoprotectants (proline, total soluble sugars and total amino acids), total phenolic-compounds and total flavonoid content were enhanced in plants co-cultivated with PGPR. Additionally, antioxidant and free radical scavenging (DPPH, hydroxyl and H_2_O_2_) activities were increased in plants that were treated with PGPR under both normal and N_2_ deficit condition. Overall, these results indicate that plants co-cultivated with PGPR, *S*. *maltophilia*, increase plant growth, antioxidant levels, scavenging, and stress tolerance under N_2_ deficit condition. The beneficial use of bacterium *S*. *maltophilia* could be explored further as an efficient PGPR for growing agricultural crops under N_2_ deficit conditions. However, a detail agronomic study would be prerequisite to confirm its commercial role.

## Introduction

Different abiotic stresses are a major problem associated with arid and semi-arid regions. Stresses are of natural or human-induced (anthropogenic) processes that inhibit plant growth [1]. Salinization is a major constraint for the crop productivity and it has been estimated that an approximate area of 7 million hectares of land is covered by saline soil in India [2]. Salinity affects the glycophytic plants at cellular, morphological, physiological and molecular levels [3, 4]. Salt interrupts the soil nutrient balance which ultimately affects the growth of plant [5]. Halophytes have the ability to grow under high saline areas and are considered a rich source of metabolites [6, 7], oligosaccharides [8], proteins [9], genes [10–22], promoters [23–25] and renewable energy [26].

The narrow region of soil that is closest to the plant root and is directly influenced by root exudates and associated-microorganisms is known as the rhizosphere. Rhizosphere is considered highly nutritive, therefore is highly competitive for soil microbes. Soil bacteria that inhabitant in the rhizosphere and enhance the plant growth are known as plant growth promoting rhizobacteria (PGPR). PGPR colonize the root surface and induce these positive effects on the plant, and act as (1) bio-fertilizers (increasing plant nutrient availability via phosphate solubilization and siderophore production), (2) phytostimulators (by promoting plant growth through phytohormones), (3) rhizo-remediators (degrading organic pollutants) and (4) biopesticides (controlling diseases through production of antibiotics, antifungal metabolites and biofilms) [27]. PGPR have enormous potential to increase crop productivity under normal as well as stressful environmental conditions.

*Stenotrophomonas* is a genus of Gram negative bacteria and belongs to Xanthomonadaceae family. A species of *Stenotrophomonas*, *S*. *maltophilia* was isolated from the rhizosphere of *Cyperus laevigatus* and demonstrated to present different bioactivities including anti-quorum sensing and antibiofilm [28], biological control of fungal plant diseases, and bioremediation [29]. *Cyperus laevigatus* is a species of sedge which grows in the coastal saline area and harbors beneficial rhizospheric bacteria such as *Delftia tsuruhatensis* and *Exiguobacterium indicum* [30, 31]. Plant growth promoting potential of *S*. *maltophilia* has been reported in wheat plants along with resistance against biotic and abiotic stress [32].

*Arachis hypogaea* (peanut) is an economically important crop which is utilized for oil, food, fiber and fodder for livestock. Peanut seeds contain approximately 40–60% oil, 20–40% protein and 10–20% carbohydrate, and many vitamins and minerals [33]. India ranks second worldwide in terms of peanut production (6–7 million tons per year) after China, but its production has declined immensely because of various environmental stresses including nitrogen deficiency in the soil. Some transgenic approaches have been employed for developing abiotic stress tolerant peanut [34–36]. However, developing a transgenic peanut is time consuming and laborious method, ethical and environmental issues make it difficult [37]. Subsequently, it is clear that an environment friendly and natural method is preferred for the enhanced productivity of crops. It has been noted that CO_2_-fixing bacterial communities were observed as part of the peanut rhizosphere which hints at the possibility of peanut-microbe interactions [38].

Nitrogen deficiency in the soil is one of the major causes that leads to low productivity and health of the crop. Reclamation of these type of soils requires the excessive application of chemical fertilizers, however, PGPR have the potential to protect plants under such conditions. In this study, we observed the interaction of *S*. *maltophilia* BJ01 with peanut plants, and effect on morphology and plant growth, changes in physiology, production of ROS, and different activities (antioxidant and scavenging) of peanut plants were analyzed under normal and nitrogen deficit conditions.

## Materials and methods

### Plant material, bacterial strain and treatment

Peanut seeds (cultivar GG-20) were obtained from the Junagadh Agricultural University, Junagadh (Gujarat), India. Dry and mature peanut seeds were washed with 70% (v/v) aqueous-ethanol followed by surface sterilization with 0.1% mercuric chloride for 10 min with gentle shaking [35]. The seeds were thoroughly washed with sterile water (five to six times) and soaked for 3 h in water. Seeds of uniform size were placed on sterilized cotton in tissue culture bottles containing ½ Murashige and Skoog (MS) media [(NH_4_)_2_NO_3_ 825 mg L^-1^; KNO_3_ 950 mg L^-1^; KH_2_PO_4_ 85 mg L^-1^; MgSO_4_.7H_2_O 185 mg L^-1^; CaCl_2_.2H_2_O 220 mg L^-1^; KI 0.41 mg L^-1^; H_3_BO_3_ 3.1 mg L^-1^; MnSO_4_.4H_2_O 11.15 mg L^-1^; ZnSO_4_.7H_2_O 4.3 mg L^-1^; CoCl_2_.6H_2_O 0.0125 mg L^-1^; CuSO_4_.5H_2_O 0.0125 mg L^-1^; Na_2_MoO_4_.2H_2_O 0.0625 mg L^-1^; FeSO_4_.7H_2_O 13.9 mg L^-1^; Na_2_EDTA 18.6 mg L^-1^; pH 5.8] for germination. Previously, we have isolated bacterial strain *Stenotrophomonas maltophilia* BJ01 from the roots of *Cyperus laevigatus* L., near costal region of Dwarka, Gujarat, India [28], and deposited at Indian marine microbial culture collection of CSMCRI, Bhavnagar with culture collection number IMMCC255. To check nitrogen fixing ability of the bacteria, nitrogen-free semisolid (NFb) medium with malate as a carbon source was used for growth. Further total DNA of the bacterium, *S*. *maltophilia* BJ01 were isolated, the *nifH* gene was amplified using degenerate primers [39]. Polymerase chain reaction (PCR) amplified products were analyzed on an agarose gel and purified using the QIAquick gel extraction kit (Qiagen, Germany). The purified PCR amplicons were cloned in pGEM-T Easy cloning vector (Promega, USA) and transformed into *Escherichia coli* DH5α competent cells. Positive clones were selected, confirmed and sequenced (M/s Macrogen Inc., South Korea).

For the bacterial inoculum preparation, the bacterial strain was streaked on DYGS (dextrose 1.0 g L^-1^; malate 1.0 g L^-1^; peptone 1.5 g L^-1^; yeast extract 2.0 g L^-1^; MgSO_4_.7H_2_O 0.5 g L^-1^; L-glutamic acid 1.5 g L^-1^; pH 6.0) agar plate and incubated for 16 hr at 30°C. Single colony from the plate was inoculated in 5 mL DYGS broth media and incubated overnight at 30°C and 180 rpm in an incubator shaker. Overnight grown culture was diluted to OD_600nm_ 0.01 in 150 mL of DYGS medium and grown up to OD_600nm_ 0.6 in an incubator shaker (30°C and 180 rpm). Freshly grown 150 mL (OD_600_ 0.6) bacterial culture was centrifuged at 4000 x *g* for 10 min. Pellet was re-suspended in 300 mL ½ MS media supplemented with or without nitrogenous component. The ½ MS media containing all macronutrients, micronutrients and vitamins is considered control media/ condition with nitrogenous source (C), whereas MS media that did not contain any nitrogenous constituents/ ingredients (such as ammonium nitrate and potassium nitrate from macronutrients, and vitamins) was considered media without nitrogenous source or nitrogen deficit media/ condition (N)

Seven days old germinated seedlings were transferred to hydroponics condition in a glass beaker containing 300 mL ½ MS media supplemented with (C) or without nitrogenous component (N). The experiment was first divided in two sets, i) control (C: control plants grown in a media supplemented with nitrogen source) and stress (N: plants grown under nitrogen deficient condition) followed by further division in two sub-sets; C–and C+ (control plants grown without or with PGPR), and N–and N+ (plants under nitrogen deficient without and with bacteria). Seedlings were transferred to the particular growth condition for twenty-one days at 25 ± 2°C temperature, 16 h/ 8 h light/dark cycle, and 170±25 μmol m^-2^ s^-1^ light intensity. Corresponding media were replenished every seven days, and different morphology characteristics including shoot length, root length and fresh weight were recorded, and images were captured for each plant. After completion of 21 days, growth characteristics, physio-biochemical properties and metabolic activities were studied.

### Fatty acid profiling

Total lipid was extracted from 300 mg plant samples (fresh leaves) using chloroform–methanol–phosphate buffer (1:2:0.9 v/v/v, pH 7.5; 10 mL), and fatty acids were converted to corresponding methyl esters (FAMEs) by transmethylation. For transmethylation, 1 mL of NaOH (1% v/v in methanol) was added, and mixture was incubated at 55°C for 15 min, after that 2 mL of methanolic HCl (5% v/v) was added and further incubated at 55°C for 15 min. Finally 3 mL of deionized water–hexane mixture (1:2 v/v) was added. FAMEs were extracted in three times in hexane, samples were pooled together and dried under vacuum. Dried sample was resuspended in 200 μl hexane and analyzed by using a RTX 5MS capillary column in GCMS-QP2010 (Shimadzu, Japan) coupled with an auto-sampler (AOC-5000) [6].

### Chlorophyll and carotenoid content

Leaf chlorophyll and total carotenoid contents were estimated according to the methods described by Arnon and Chamovitz *et al*. [40, 41]. Briefly, leaf tissues (100 mg) were homogenized in 80% acetone, incubated for 6hrs in the dark, centrifuged at 10000 x *g* and absorbance of supernatant was recorded at 461, 645, 663, and 664nm. Total carotenoid and chlorophyll contents were calculated using the following equations:

$$Total\;Chlorophyll=\frac{[\left(20.2\times {Abs}_{645})+(8.02\times {Abs}_{663}\right)]\times vol\;of\;sample\;in\;ml}{weight\;of\;tissues}$$

$$Chlorophyll\;a=\frac{[\left(12.7\times {Abs}_{663})-(2.6\times {Abs}_{645}\right)]\times vol\;of\;sample\;in\;ml}{weight\;of\;tissues}$$

$$Chlorophyll\;b=\frac{[\left(22.9\times {Abs}_{645})-(4.68\times {Abs}_{663}\right)]\times vol\;of\;sample\;in\;ml}{weight\;of\;tissues}$$

$$Total\;carotenoid=[\left({Abs}_{461})-(0.046\times {Abs}_{664}\right)]\times 4$$

### Electrolyte leakage

Leaves of equal size and age were harvested from primary branch (toward the distal end) of each experimental plant and washed thoroughly with deionized water to remove surface-adhered electrolytes. Samples were kept in deionized water (10 mL) and incubated at 25°C on a rotary shaker for 24 h. The electrical conductivity (EC) of the solution (L_1_) was determined using a conductivity meter (Seven Easy, Mettler Toledo, USA). Samples were autoclaved at 120°C for 20 min, cooled at 25°C, and electrical conductivity (L_2_) was determined [42]. The electrolyte leakage was estimated with the following equation:

$$EL(\%)=\frac{L_{1}}{L_{2}}\times 100$$

### Membrane stability index

To determine the membrane stability index (MSI), thoroughly washed leaf samples (equal size and age) were kept in 10 mL deionized water, incubated at 40°C for 30 min, and EC (L_1_) was recorded. Samples were boiled at 100°C for 20 min, then they were cooled at 25°C, and EC (L_2_) was recorded to calculate MSI [43]. Following equation were used for the calculation:

$$MSI=\left[1-\frac{L_{1}}{L_{2}}\right]\times 100$$

### Lipid peroxidation

Lipid peroxidation was determined by quantifying the malondialdehyde (MDA) content according to method described by Hodges *et al*. [44]. In brief, leaf samples (100 mg) were homogenized in liquid nitrogen and extracted. In one set of reaction, leaf extract was mixed with an equal volume of thiobarbituric acid reagent containing thiobarbituric acid (TBA) and trichloroacetic acid (TCA) (TBA; 1 mL of 0.5% w/v prepared in 20% w/v TCA). In another set of reaction, extract was mixed with an equal volume of TCA (20% w/v). Reaction mixtures were incubated at 95°C for 30 min, cooled at 25°C, and centrifuged at 10000 x *g* for 5 min. Absorbance of the supernatant was recorded at 440 nm, 532 nm, and 600 nm. MDA content was quantified using the following equation:

$$A=[{Abs}_{532+TBA}-{Abs}_{600+TBA}]-[{Abs}_{532-TBA}-{Abs}_{600-TBA}]$$

$$B=[{Abs}_{440+TBA}-{Abs}_{600-TBA}]\times 0.0571$$

$$MDA\;(\mu mol\;g^{-1})=\frac{A-B}{15700}\times 10^{6}$$

### Total H_2_O_2_ content

Leaf samples (100 mg) were extracted in 80% cold acetone and hydrogen peroxide was determined by the modified method described by Mukherjee and Choudhuri [45]. Absorbance was measured at 415 nm. Total H_2_O_2_ content of samples was calculated by a standard curve drawn with the known concentration of H_2_O_2_.

### Auxin content

For the quantification of auxin contents, Leaf samples were homogenized in liquid nitrogen and extracted with 95% ethanol. Colorimetric assay was performed with Salkowski reagent and the absorbance was recorded at 535nm [46].

### Proline content

Free proline contents of harvested leaf samples were quantified by acid ninhydrin reagent as described by Bates et al. [47]. One hundred mg plant leaves were homogenized in liquid nitrogen and extracted in aqueous sulphosalicylic acid. An equal volume of the extract and the acid ninhydrin reagent are mixed together and incubated at 100°C for 1 h. Reaction was terminated by cooling the sample in an ice bath. Toluene was added after cooling the sample mix, vortexed, and upper phase was aspirated to measure the absorbance at 520nm. Total proline content was calculated using a standard curve of known concentration of proline.

#### Total amino acid content

Total amino acid content of plant samples was determined by previously described method [48]. Plant leaf samples (100 mg) were extracted with 80% ethanol, and extract was treated with an equal volume of 0.2 M citrate buffer (pH 5) along with ninhydrin reagent (1% ninhydrin). The reaction mixture was incubated at 95°C in a water bath for 15 min. Samples were cooled to room temperature centrifuged and the absorbance was read at 570 nm.

#### Total soluble sugars

Total soluble sugar contents were calculated according to the previously described method [49]. One hundred microgram leaf samples were homogenized with liquid nitrogen and extracted in 1 mL of 80% ethanol. Three milliliter freshly prepared anthrone reagent (150 mg anthrone in 100 mL of 72% v/v H_2_SO_4_) was added to 100 μL extract, kept at 100°C in water bath for 10 min. Reaction mixtures were cooled at room temperature and the absorbance was measured at 625 nm

#### Extract preparation for the analysis of metabolic activities

Five gram leaves were harvested from control and treated plants, powdered by homogenizing in liquid N_2_, and added to the aqueous methanol (70% v/v). After 16 hr of incubation sample were centrifuged at 10000 x *g* for 10 min, supernatant was collected in fresh reagent bottle and aqueous methanol was again added to the sample for re-extraction. After double extraction supernatant was pooled, concentrated under vacuum using a rotary evaporator (Büchi, Switzerland), and lyophilized at -80°C (VirTis Sentry, USA) and stored at -20°C until further use.

#### Total phenolic content

Total phenolic content of the samples was estimated by the Folin–Ciocalteu reagent. The Plant extract was added in 2.5 mL Folin–Ciocalteu reagent (0.2M; Sigma-Aldrich, USA) mixed and incubated at room temperature. After 5 min of incubation, 2 mL sodium carbonate (Na_2_CO_3_; 75 g L^-1^) were mixed in the reaction mixture and incubated in dark at room temperature for 90 min. The absorbance was measured at 760 nm, and the total phenolic content was calculated as gallic acid equivalent (GAE) from a standard curve plotted with the known concentration of gallic acid [50, 51].

#### Total flavonoid content

Total flavonoid content was measured as described by Zhishen *et al*. [52]. Plant extracts were mixed with NaNO_2_ (5% w/v), incubated at room temperature for 5 min, followed by addition of AlCl_3_ (10% v/v). After 6 min, 1M NaOH was added to reaction mixture, mixed well by vortex and absorbance was measured at 510 nm. The total flavonoid content was calculated from a standard curve of quercetin.

#### Total antioxidant activity

Total antioxidant activity was measured by 2,2′-azino-bis (3-ethylbenzothiazoline-6-sulphonic acid) free radical (ABTS^+^) scavenging ability of the extracts of To generate the free radicals, ABTS diammonium salt (7 mM) solution was mixed with potassium persulfate (2.45 mM), and incubated overnight in the dark at room temperature. After the generation of stable free radicals, absorbance of ABTS^+^ radical solution was adjusted to A_734nm_ = 0.70 ± 0.02 and equilibrated at 30°C. Different concentrations of the extract (10–50 μg mL^-1^) or the standard (1–5 μg mL^-1^) were added to the ABTS^+^ radical solution and absorbance was measured at 734 nm after 5 min. Trolox was used as standard and percentage inhibition of absorbance was calculated [53].

#### DPPH free radical scavenging assay

To check the free radical scavenging of extract, 2,2′- diphenyl-1-picrylhydrazyl (DPPH) was used as free radical. The DPPH solution (0.024% w/v) was prepared in methanol and absorbance was adjusted to Abs_517 nm_ 0.98 ± 0.02 using methanol. Different concentrations of extracts (10–80 μg mL^-1^) were mixed in DPPH solution (Abs_517 nm_ 0.98 ± 0.02) and incubated for 15 min at room temperature in the dark. The absorbance was measured at 517 nm and the radical scavenging activities were estimated [54].

$$Scavenging\;(\%)=\frac{{Abs}_{control}-{Abs}_{sample}}{{Abs}_{control}}\times 100$$

#### Reducing power assay

To check reducing capacity different concentrations of the plant extracts (100–1000 μg mL^-1^) were mixed with 1 mL phosphate buffer (0.2 M, pH 6.6). Thereafter 1 mL of K_3_Fe(CN)_6_ (10 mg mL^-1^) was added to the reaction and incubated at 50°C in water bath (Julabo, Germany). After 20 min of incubation, 1 mL trichloroacetic acid (100 mg L^-1^) was added to terminate the reaction. Reaction mixtures were cooled at room temperature, centrifuged at 7000 x *g* for 10 min and the supernatant was collected. In the next step 1 mL supernatant was mixed with 0.2 mL freshly prepared FeCl_3_ (0.1% w/v), incubated for 10 min at room temperature, absorbance was measured at 700 nm. Ascorbic acid was used as standard [49, 51].

#### Hydrogen peroxide scavenging activity

The hydrogen peroxide scavenging activity of different concentration of plant extracts was evaluated by previously described method [6, 48, 49, 51]. Plant extracts (0.1–0.5 mg mL^−1^) were mixed with 0.4 mL phosphate buffer (50 mM, pH 7.4) and 43 mM hydrogen peroxide (0.6 mL; prepared in phosphate buffer) added to the reaction mixture, and absorbance was recorded at 230 nm (T1). After 10 min incubation, absorbance of reaction mix was recorded at 230 nm (T2) and scavenging activity was calculated using following formula:

$$H_{2}O_{2}\;scavenging\;activity\;(\%)=\left[1-\frac{{Abs}_{sample\;at\;T2}}{{Abs}_{sample\;at\;T1}}\right]\times 100$$

#### Hydroxyl radical scavenging assay

Hydroxyl radical scavenging activity was performed with different concentrations (10–100 μg) of plant extracts using Fenton reaction (Fe^3+^-ascorbate-EDTA-H_2_O_2_) as described by Saeed *et al*. [54]. Plant extracts were mixed with 500 μL of 2.8 mM 2-deoxyribose prepared in 50 mM potassium phosphate buffer (pH 7.4). Thereafter, 200 μL of 100mM FeCl_3_ and 100mM EDTA solution (1:1 v/v) and 100 μL of 200 mM H_2_O_2_ were added to reaction mixture. Reaction was started by adding 100 μL of 300mM ascorbic acid to the reaction mixture and incubated for 1 h at 37°C. After incubation, 500 μL reaction mixture was added to the 1 mL of TCA solution (2.8% w/v) followed by addition of 1 mL of aqueous TBA solution (1% prepared in 0.025 M NaOH containing 0.02% BHA) and incubated at 99°C in water bath (Julabo, Germany) for 15 min. Reactions were cooled at room temperature and absorbance was recorded at 532 nm. The following formula was used to calculate percent scavenging activity.

$$Hydroxyl\;scavenging\;activity\;(\%)=\left[1-\frac{{Abs}_{sample}}{{Abs}_{control}}\right]\times 100$$

### Statistical analysis

Statistical analysis was performed by GraphPad Prism software. One-way ANOVA followed by Tukey post-hoc test was applied to compare the test and controls. Values are expressed as the mean ± SE, and statistically significant differences are marked with different stars.

## Results and discussion

### Nitrogen fixing ability of *Stenotrophomonas maltophilia* BJ01

The bacterium *S*. *maltophilia* was grown in nitrogen-free semisolid NFb medium with malate as a carbon source to confirm the nitrogen-fixing ability of the bacterial strain. Further, an amplicon of expected 360 bp was obtained with degenerate *nifH* primers [55, 56], which confirmed the presence of the *nifH* gene in the bacterium (S1 Fig). The sequence analysis showed 99% query coverage and 99.44% homology with uncultured bacterium dinitrogenase reductase (*nifH*) gene (JN162497) and also showed 99% query coverage and 83.29% homology with *nifH* gene of culturable bacterium *Bradyrhizobium japonicum* (GQ289567). The *nifH* gene sequence of *S*. *maltophilia* BJ01 was submitted to NCBI (GenBank: JX545230).

### *Stenotrophomonas maltophilia* BJ01 alters the plant fatty acid composition

Fatty acid composition of peanut seedling was highly influence by the interaction with *S*. *maltophilia* (Table 1). Under control condition (with N_2_ source), about 84.75% heptadecenoic acid was detected followed by hexadecanoic acid (6.74%) and pentadecenoic acid (6.6%), whereas other fatty acids were negligible. In contrast, heptadecenoic acid was not detected when plants were grown with nitrogenous source and bacterium. Furthermore, hexadecanoic acid (37.5%) detected utmost followed by heptadecanoic acid (33.43%) and 6,9,12-octadecatrienoic acid (28.62%). Under N_2_ deficit condition, the maximum content of heptadecanoic acid (76.54%) was detected in control plants (without N_2_ source) followed by hexadecanoic acid (11.65%) and 6,9,12-octadecatrienoic acid (8.07%). High content of tetradecanoic acid (C14:0; 41.11%) was observed in the plants grown under N_2_ deficit condition along with bacterial inoculum, followed by 9-hexadecenoic acid (30.15%), hexadecanoic acid (15.94%), pentadecanoic acid (9.26%) and 6,9,12-octadecatrienoic acid (2.16%). A change in the fatty acid composition was observed due to interaction between *S*. *maltophilia* and peanut under control and stress condition.

**Table 1:** Fatty acid composition of peanut plants grown under control or N_2_ stress conditions with or without bacteria *Stenotrophomonas maltophilia* BJ01.

FAs	Fatty acid	Control (with nitrogen)		Stress (without nitrogen)	
		C– without bacteria	C+ with bacteria	N‒ without bacteria	N+ with bacteria
C12:0	Dodecanoic acid	nd	nd	nd	0.12%
C13:0	Tridecanoic acid	nd	nd	nd	0.10%
C14:0	Tetradecanoic acid	0.43%	nd	nd	41.11%
C15:0	Pentadecanoic acid	nd	nd	0.18%	9.26%
C15:1	10-Pentadecenoic acid	6.60%	nd	nd	0.12%
C16:0	Hexadecanoic acid	6.74%	37.50%	11.65%	15.94%
C16:1 (cis-9)	9-Hexadecenoic acid	nd	0.07%	0.51%	30.15%
C17:0	Heptadecanoic acid	0.02%	33.43%	76.54%	0.51%
C17:1	10-Heptadecenoic acid	84.74%	nd	0.41%	0.53%
C18:0	Octadecanoic acid	nd	0.01%	nd	nd
C18:1 (trans-9)	9-Octadecenoic acid	0.04%	nd	0.17%	nd
C18:2 (cis-9,12)	9,12-Octadecadienoic acid	0.36%	0.37%	0.17%	nd
C18:3 (cis-6,9,12)	6,9,12-Octadecatrienoic acid	0.49%	28.62%	8.07%	2.16%
C18:3 (cis-9,12,15)	9,12,15-Octadecatrienoic acid	0.58%	nd	2.31%	nd

nd: not detected or negligible amount detected. Control (C) and stressed (N) peanut seedlings (seven days old) grown in hydroponics (Hoagland solution) with (C+ and N+) or without (C–and N–) bacterial inoculum for 21 days

Peanut is an edible oil-yielding plant and grown worldwide for commercial edible-oil production. Its fatty acid composition is considered beneficial for human health and widely used in the human diet. It was observed that PGPR *S*. *maltophilia* interaction altered fatty acid composition while interacting with peanut plants under both normal and N_2_ stress conditions (Table 1). Palmitoleic acid or 9-Hexadecenoic acid is an omega-7 monounsaturated fatty acid, which is biosynthesized from palmitic acid, and an enhanced concentration was detected in plants grown with PGPR under N_2_ stress condition. Monounsaturated fats are well known to provide membrane fluidity and thus protect against cardiovascular disease. Similarly, 6,9,12-Octadecatrienoic acid, also known as Gamma-linolenic acid or GLA (γ-Linolenic acid) is an omega-6 fatty acid, and its concentration increased after PGPR interaction. GLA has been reported to reduce atopic dermatitis in a double-blind, placebo-controlled clinical trial [57]. It was also noticed that content of mono-saturated fatty acids increased in plants during PGPR inoculation. It was shown that saturated fatty acid has no effect on blood cholesterol levels [58] whereas some saturated fatty acids have antibacterial activity [59]. Similar to this study, inoculation of PGPR *Bradyrhizobium japonicum* altered the fatty acids composition of soybean [60]. It was established that fatty acids content regulates the cell-membrane fluidity, and therefore alleviates the plant tolerance to different stress condition [61]. Surprisingly, Cagide *et al*. [62] did not find any change in the fatty acid composition of Soybean grown with *Bradyrhizobium elkanii* and *Delftia* sp. Strains. A similar result was also observed with alfalfa plants cocultivated with *S*. *meliloti* [63]. It was speculated that inoculation of PGPR *S*. *maltophilia* may have altered the fatty acid composition of peanut plants, resulted in the improved plant-tolerance to N_2_ deficit condition by modulating membrane fluidity.

### Plant growth and photosynthetic pigments are influenced by *S*. *maltophilia* BJ01

**Fig 1:**
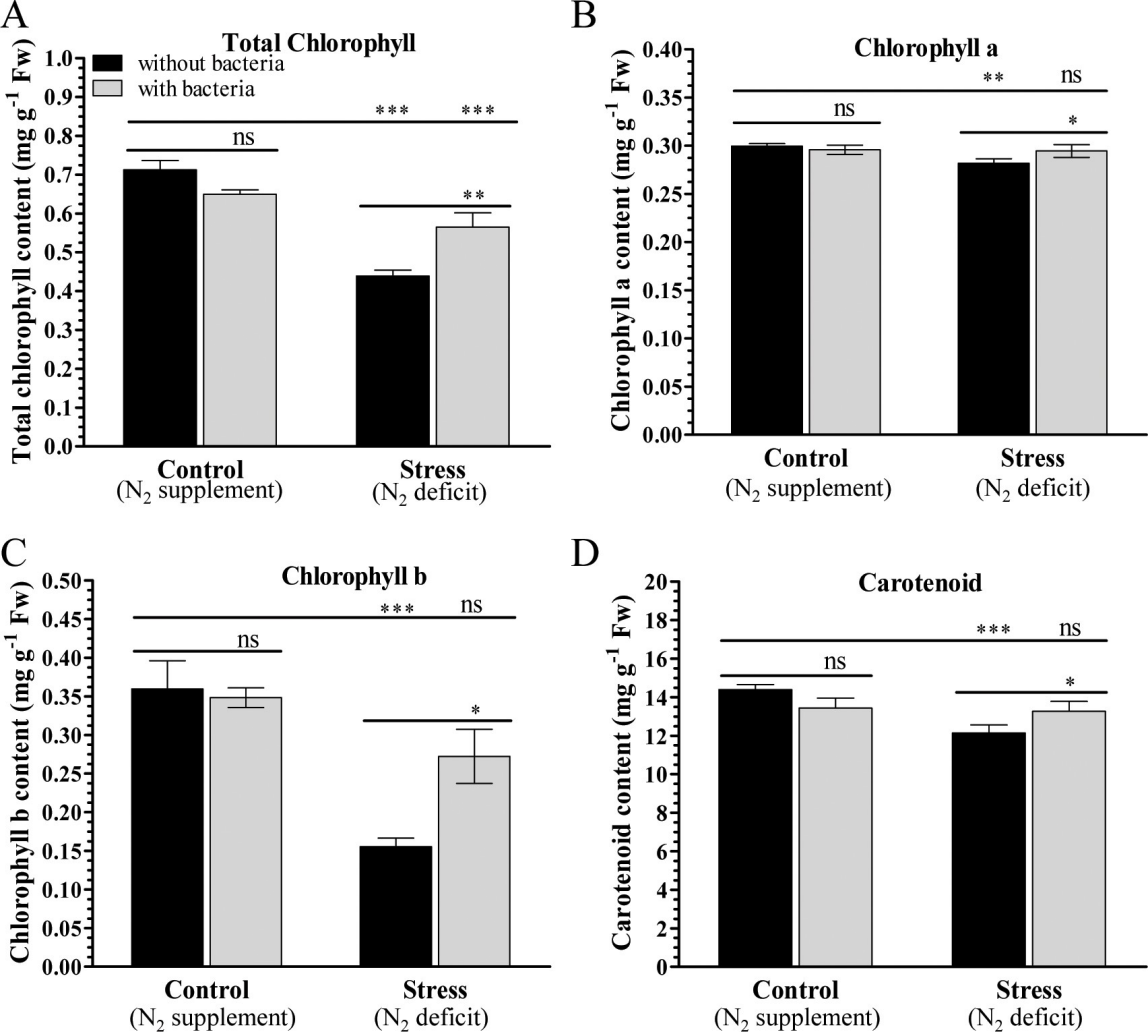
Chlorophyll content and carotenoids content of control and stressed peanut plants grown with or without bacterium. (A) Total chlorophyll, (B) chlorophyll a, (C) chlorophyll b, and (D) carotenoids contents of peanut plants grown under nitrogen supplement (C) or nitrogen deficit (N) conditions with inoculum (C+ and N+) or without inoculum (C–and N‒). Bars represent means ± SE, and ‘*’, ‘**’ and ‘***’ indicate significant differences at *P* < 0.05, *P* < 0.01 and *P* < 0.001, respectively, while ‘ns’ means no significant difference.

The total chlorophyll, (about 0.7 mg g^−1^ Fw), Chl a (about 0.3 mg g^−1^ Fw), Chl b (about 0.4mg g^−1^ Fw), and carotenoid (about 14 μg g^−1^ Fw) content were comparable between control plants (media supplemented with nitrogenous source) grown with (C+) or without (C–) bacterial inoculum (Fig 1). Under N_2_ deficit conditions, chlorophyll (total, a and b) and carotenoid contents decreased in stressed plants grown without bacteria (N–) compared to control plants (Fig 1). About 0.4, 0.25, 0.15 and 0.12 mg g^−1^ Fw total chl, chl a, chl b and carotenoid contents were estimated in stress plants grown without bacteria. It was observed that bacterial inoculation enhances the photosynthetic pigments of plants under N_2_ deficit conditions. Higher amount of total chlorophyll (about 0.6), chl a & b (about 0.3), and carotenoid (about 14 mg g^−1^ Fw) were detected in stress plants (N+) grown with bacterial inoculum compared to those plants grown without bacteria under N_2_ stress condition.

**Fig 2:**
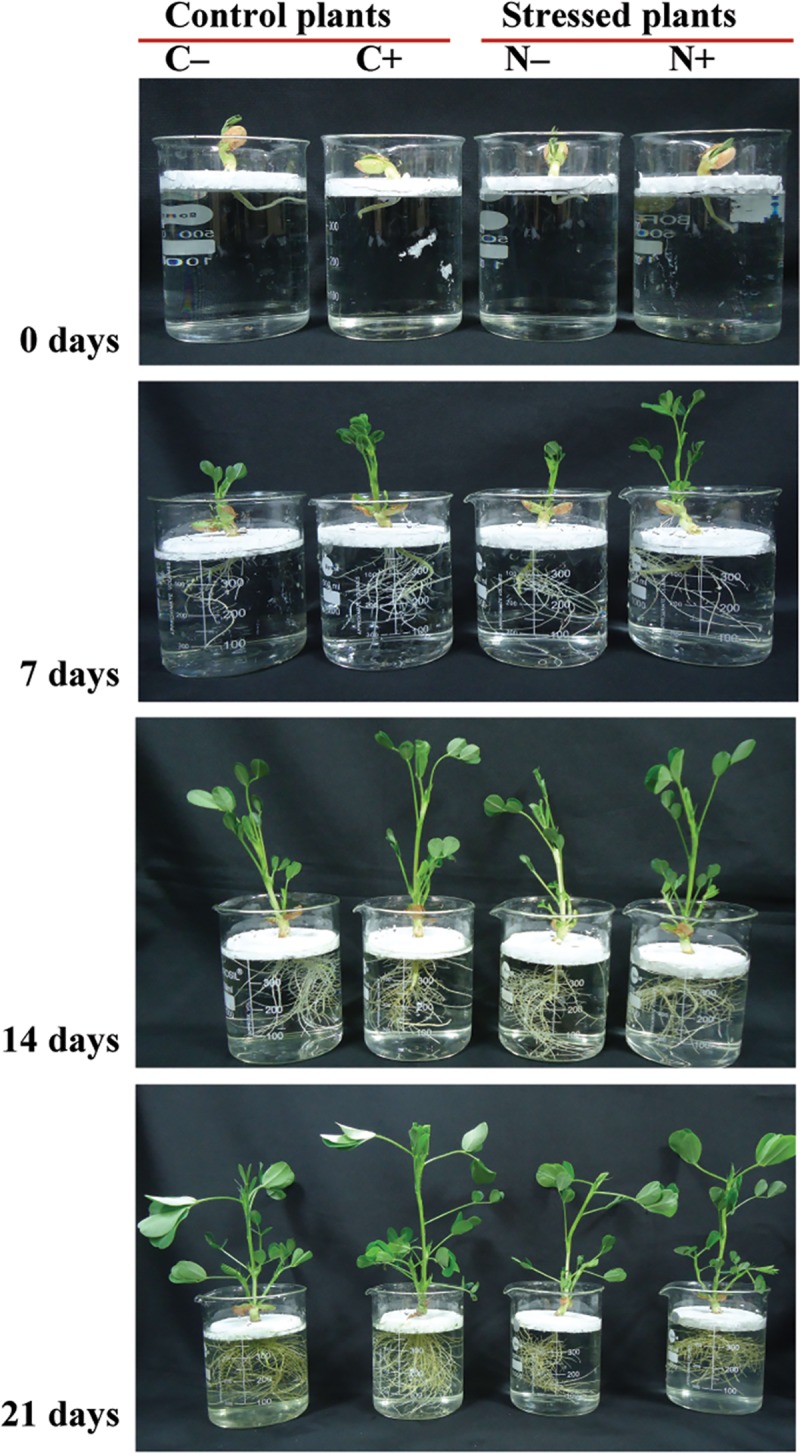
Morphology of control and stressed peanut plants grown with or without bacterial inoculum. Control (C: medium supplemented with nitrogenous source) and stressed (N: nitrogen deficit condition) peanut seedlings grown in hydroponics (Hoagland solution) with (C+ and N+) or without (C–and N–) bacterial inoculum for 21 days.

PGPRs are widely used in agriculture for enhanced growth and productivity of crops, and the most common beneficial bacteria are *Azospirillum* spp. and rhizobia [64]. It is hypothesized that PGPRs influence the content of photosynthesis pigments, and thus control the plant growth and yield. Growth characteristics of the control and treated plants did not show significant changes (S2 Fig), however, plants grown with bacterium inoculum showed dense root morphology (Fig 2). Overall, plants grown with bacterium showed better morphology (overall plant growth e.g. plant height–shoot and root length, and number of leaves) compared to their corresponding control plants (Fig 2). Chlorophylls and carotenoids are pigments which are involved in photosynthesis, they absorb light and provide the energy [65]. They are also involved in the regulation of plant growth [66]. Our results suggest that bacterial inoculation promotes plants to grow under control and stress conditions compared to corresponding plants grown without bacteria (Figs 1 and 2). Previously, an increase in the content of photosynthetic pigments was observed in wheat and *Arabidopsis thaliana* plants by inoculation with *Azospirillum brasilense* [67, 68].

### Interaction of *S*. *maltophilia* regulates the physiology and biochemical status of *Arachis hypogea*

**Fig 3:**
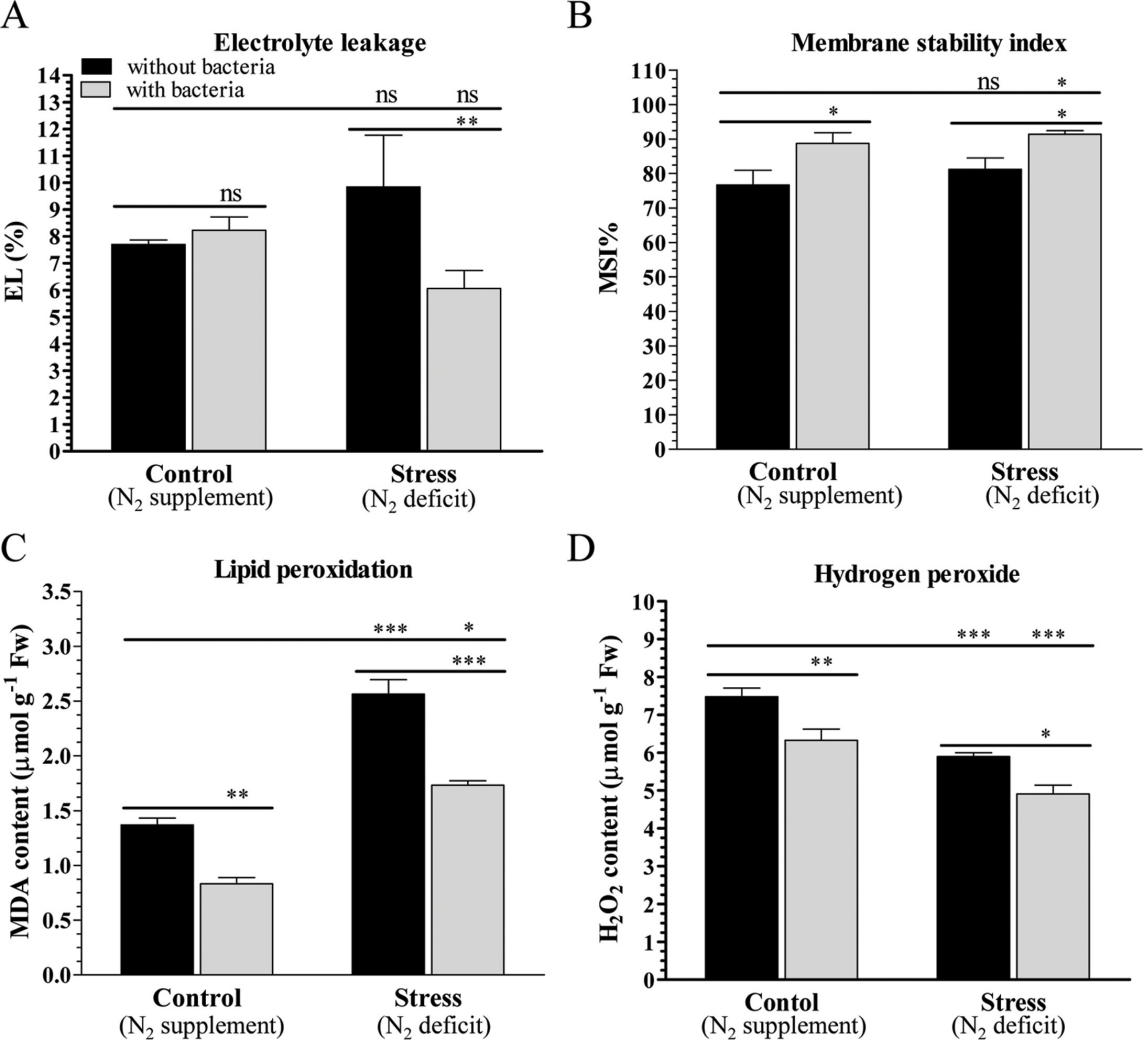
Physiological analyses of control and stressed peanut plants grown with or without bacterium. Estimation of (A) electrolyte leakage, (B) membrane stability index, (C) lipid peroxidation, and (D) hydrogen peroxide content of peanut plants grown under nitrogen supplement (C) or nitrogen deficit (N) conditions with inoculum (C+ and N+) or without inoculum (C–and N‒). Bars represent means ± SE, and ‘*’, ‘**’ and ‘***’ indicate significant differences at *P* < 0.05, *P* < 0.01 and *P* < 0.001, respectively, while ‘ns’ means no significant difference.

Electrolyte leakage, membrane stability, lipid peroxidation and hydrogen peroxide production were measured for control and stressed plants, grown with or without bacteria (Fig 3). No significant electrolyte leakage was observed in the control plants (C) grown with (C+; 8.2%) or without (C–; 7.7%) bacteria. However, under nitrogen deficit condition, electrolyte leakage decreased significantly in the plants grown with bacteria (N+; 6%) compared to those that grown without bacteria (N–; 9.8%). Similarly, lower H_2_O_2_ content was estimated in the treated plants (C+ and N+; grown with bacteria) compared to plants grown without bacterial inoculum (C–and N–). About 7.4 μmol g^-1^ Fw H_2_O_2_ were measured in the plants grown without bacteria (C–), which decreased significantly to 6.3 μmol g^-1^ Fw in the plants grown with bacteria (C+). Under nitrogen stress condition, H_2_O_2_ production further decreased significantly, and 5.5 and 4.4 μmol g^-1^ Fw H_2_O_2_ were estimated in the plants grown without (N–) or with bacteria (N+). In contrast, lipid peroxidation (measured by MDA content which is a product of lipid peroxidation and accumulated in the cells) increased under N_2_ stress condition but a quenching effect (mitigation of lipid peroxidation) was observed when plants were grown with bacteria under both normal and N_2_ stress condition. About 1.36 (C–) and 2.56 (N–) μmol g^-1^ Fw MDA contents were measured in control and N_2_ stressed plants grown without bacteria, which reduced to 0.83 (C+) and 1.73 (N+) μmol g^-1^ Fw in the plants grown with bacteria inoculum. The membrane stability indices (0.8–0.9) were almost similar for control and N_2_ stressed plants grown with or without bacteria inoculum.

**Fig 4:**
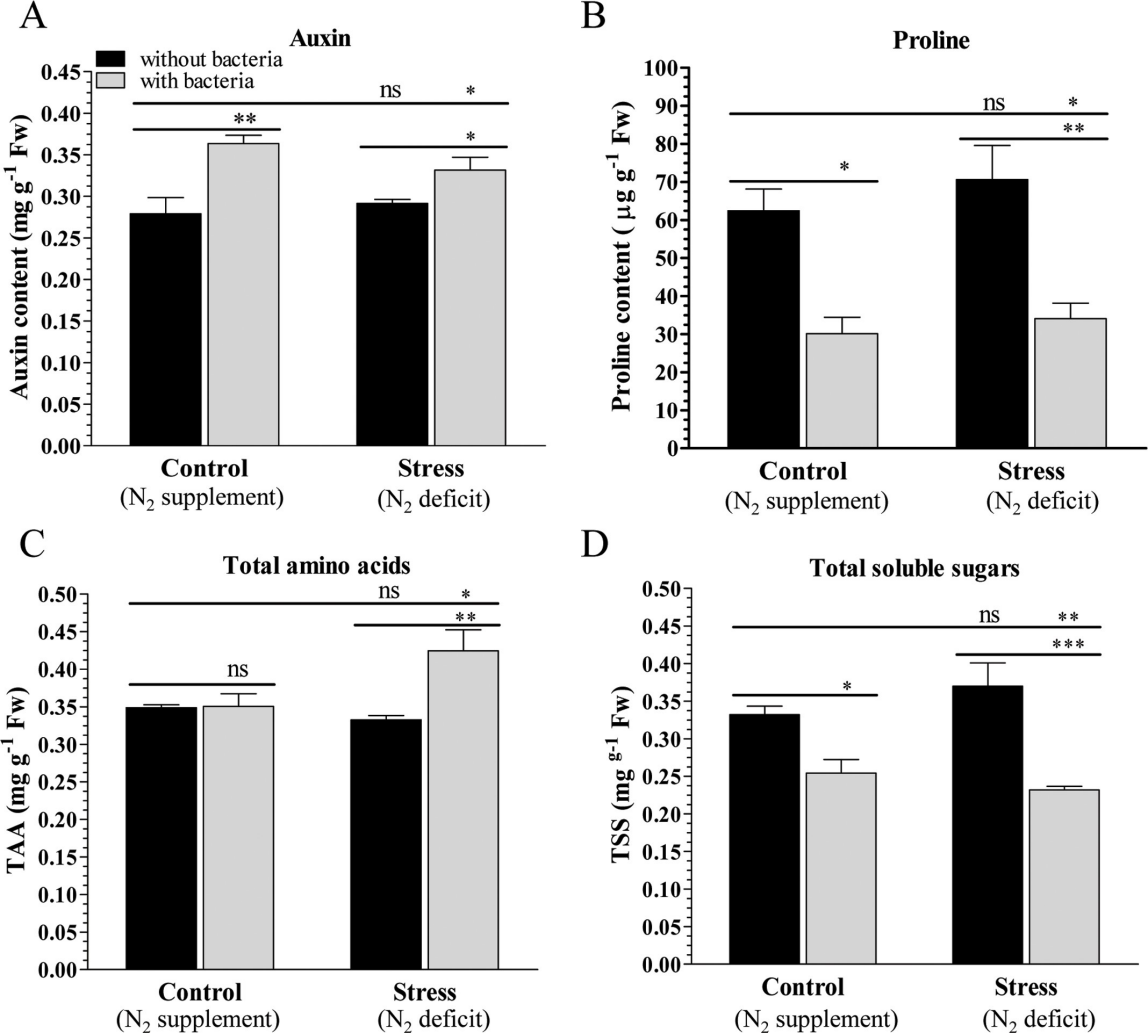
Biochemical analyses of control and stressed peanut plants grown with or without bacterium. Estimation of (A) Auxin, (B) proline, (C) total amino acids, and (D) total soluble sugars in peanut plants grown under nitrogen supplement (C) or nitrogen deficit (N) conditions with inoculum (C+ and N+) or without inoculum (C–and N‒). Bars represent means ± SE, and ‘*’, ‘**’ and ‘***’ indicate significant differences at *P* < 0.05, *P* < 0.01 and *P* < 0.001, respectively, while ‘ns’ means no significant difference.

The biochemical status of plants was studied by measuring auxin, proline, total amino-acids and total sugar contents of control and N_2_ stressed plants grown with or without bacteria inoculum (Fig 4). Auxin content was increased significantly in the plants grown with bacteria inoculum, about 0.28 mg g^-1^ Fw auxin was detected in control and N_2_ stressed plants (C–and N–) grown without bacteria which reached to about 0.4 mg g^-1^ Fw in the plants grown with bacteria inoculum. In contrast, lower proline accumulation was observed in the plants grown with bacteria inoculum compared to corresponding plants grown without bacteria. About 62 and 71 μg g^-1^ Fw proline were estimated in the control and N_2_ stressed plants grown without bacteria. Proline content was decreased more than 50%, and about 30 and 34 μg g^-1^ Fw proline were detected in the plants grown with bacteria inoculum. Similarly, total soluble sugar (TSS) was decreased in the plants grown with bacteria compared to plants grown without bacteria under both control and N_2_ stress condition. About 0.35–0.37 mg g^-1^ Fw TSS was observed in control and N_2_ stressed plants grown without bacteria, which decreased significantly to 0.25–0.23 mg g^-1^ Fw when plants were grown with bacteria inoculum. No significant difference was found in the total amino-acid (TAA) content, and about 0.35 mg g^-1^ Fw TAA was observed in all plants, however TAA increases significantly to about 0.43 mg g^-1^ Fw in the stressed plant grown with bacteria.

Accumulation of osmoprotectants such as proline, total amino acids and total sugars can protect plants and scavenge the free hydroxyl radicals [69]. Rhizobacterium *Enterobacter cloacae* is reported to protect plants under biotic and abiotic stress conditions [1]. In the present study, plants co-cultivated with PGPR *S*. *maltophilia* showed improved physiology and biochemical status under normal as well as N_2_ stress condition (Figs 3 and 4). Environmental stress promotes the generation of ROS which leads to enhanced lipid peroxidation, assessed by observation of an increase in the MDA contents [70, 71]. The decrease in MDA content confirms that bacterial inoculation protects peanut plants under control and stress conditions. Higher accumulation of auxin in inoculated plants further supports the plant growth due to PGPR under normal as well as stress condition.

### Bacterium interaction influences the antioxidant and scavenging activities of *Arachis hypogea*

**Fig 5:**
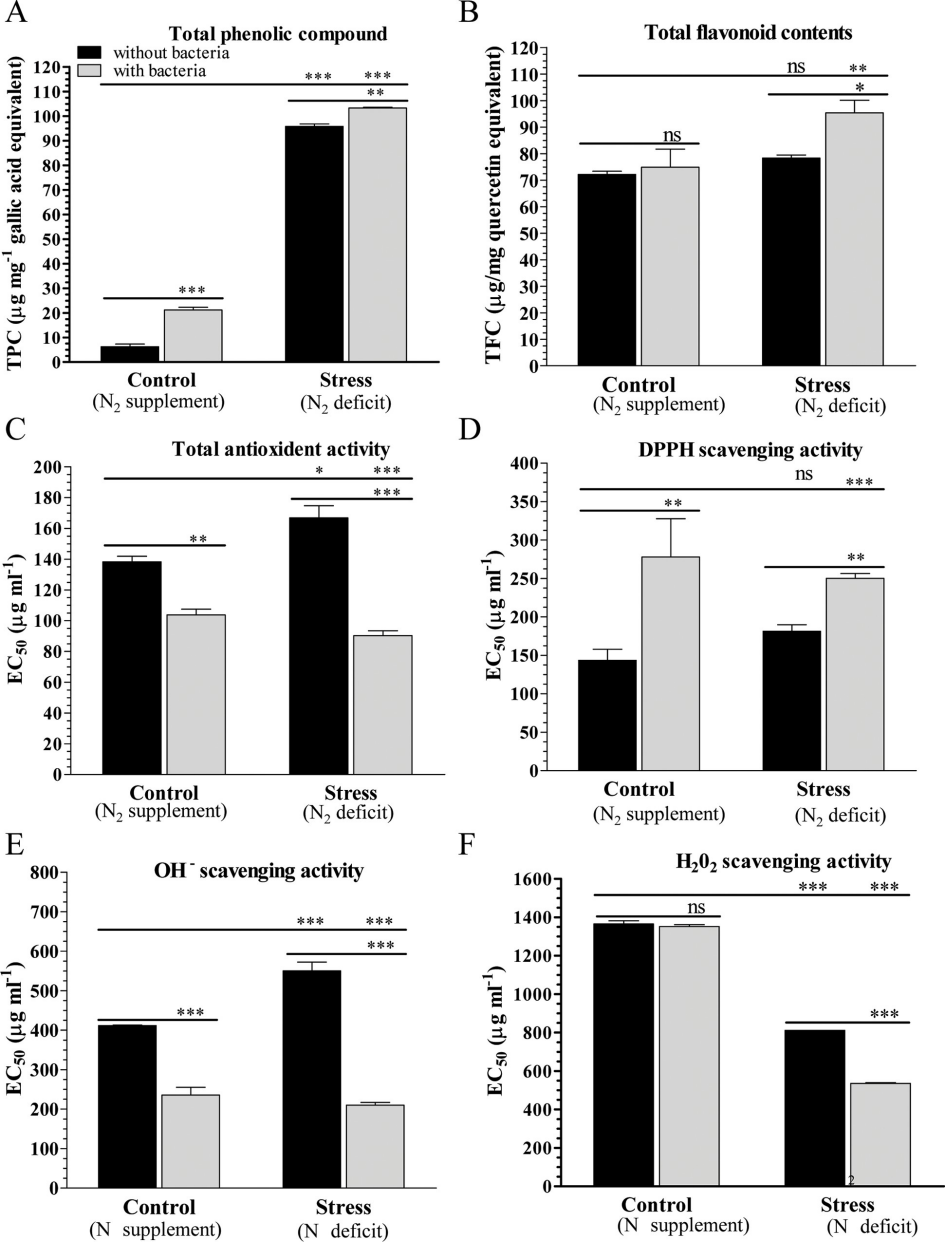
Total phenolic and flavonoid contents, and half maximal effective concentration (EC_50_) of different activities. Estimation of phenolic (TPC) and flavonoid (TFC) contents, and half maximal effective concentration of different activities (total antioxidant and scavenging–DPPH, H_2_O_2_ and OH^−^and reducing) in peanut plants grown under nitrogen supplement (C) or nitrogen deficit (N) conditions with inoculum (C+ and N+) or without inoculum (C–and N‒). Bars represent means ± SE, and ‘*’, ‘**’ and ‘***’ Indicate significant differences at *P* < 0.05, *P* < 0.01 and *P* < 0.001, respectively, while ‘ns’ means no significant difference.

Total phenolic (TPC) and total flavonoid (TFC) contents were increased in the plants when grown with bacteria. About 6.24 μg mg^-1^ gallic acid equivalent (GAE) TPC was estimated in the control plants grown without bacteria which increased significantly after bacterial inoculation (21.29 μg mg^-1^ GAE). Under N_2_ stress condition, about 95.76 μg mg^-1^ GAE TPC was measured in the plants grown without bacteria, which further increased and reached maximum (103.25 μg mg^-1^ GAE) when grown with bacterial inoculum (Fig 5). Similarly, total flavonoid content was increased and maximum content (95.35 μg QE) was estimated in the plants grown under N_2_ deficit condition with bacterial inoculum (Fig 5). Plants co-inoculated with *S*. *maltophilia* showed higher content of phenolic-compounds and total flavonoids under normal and stress condition compared to corresponding controls. These results suggest that peanut plants modify their metabolism in response to bacterium inoculum and thus produce a higher amount of TPC and TFC. Further, total phenolic-compounds and total flavonoids influence the plant defence against free radicals under normal and N_2_ stress condition.

The N_2_ deficit condition may lead to free radical formation during nitrogen fixation, therefore different antioxidant and scavenging activities were studied under normal and stress condition. Total antioxidant and scavenging (DPPH, hydrogen peroxide and hydroxyl ions) activities were found concentration dependent (S3 Fig). Total antioxidant and scavenging activities were increased concomitantly with the increasing concentration of plants extracts, and maximum activity (except DPPH scavenging) was noticed for the N_2_ stressed plants grown with bacterial inoculum (N+). In contrast, maximum DPPH scavenging activity was observed with stressed plant extracts grown without bacterial inoculum (S3 Fig).

The half maximal effective concentration (EC_50_) was estimated for the different antioxidant and scavenging activities (Fig 5). The EC_50_ for total antioxidant was lower for stressed plants grown with bacteria (N+; 90 μg mL^-1^) compared to control plants grown with bacteria (C+; 104 μg mL^-1^), followed by control plants grown without bacteria (C–; 139 μg mL^-1^) and stressed plants grown without bacteria (N‒; 167 μg mL^-1^). Similarly, for hydroxyl ions scavenging activity, the lowest EC_50_ of 210 μg mL^-1^ was detected for N+, followed by C+ (236 μg mL^-1^), C–(412 μg mL^-1^) and N‒ (551 μg mL^-1^). Plants grown under N_2_ deficit condition showed maximum H_2_O_2_ scavenging activity compared to plants grown under control condition. In contrast, a decrease in the DPPH scavenging activity was observed in plants after bacterial interaction under both control and stress condition. In addition bacterial interaction showed maximum antioxidant and scavenging activities that required the lowest EC_50_ dose (Fig 5).

Phenolic, flavonoids and secondary metabolites are plant-derived compounds which play a key role in defence under stress condition [72]. Phenolic-compounds are considered precursors of several signalling molecules which are involved in plant growth, redox reactions and stress tolerance [73, 74]. In legumes, flavonoids (like daidzein and genistein) and coumestrol trigger the nodule formation [75, 76]. In this study, an enhanced total phenolic and flavonoid contents were detected in plants which are co-cultivated with bacterium inoculum (Fig 5).

Generation of free radicals lead to auto-oxidation, and maintain oxidation/reduction equilibrium which is important for plant growth and stress tolerance [77]. TPC and TFC are known for their antioxidant and scavenging potential [78], as well as our results showed that PGPR *S*. *maltophilia* improves the antioxidant and scavenging activities of peanut plants by enhancing the TPC and TFC. Similarly, soybean plants inoculated with PGPR also enhanced antioxidant and scavenging activities [59, 79, 80].

## Conclusion

In the study, the PGPR effect of *Stenotrophomonas maltophilia* BJ01 strain was evaluated by co-cultivating with *Arachis hypogaea* GG20 (peanut) plants under normal and nitrogen deficient conditions. Plants use metabolites, especially secondary metabolites (alkaloids, flavonoids and phenolics), as their defence system in the face of various biotic and abiotic stresses. Earlier studies have shown the changes in metabolic profile and essential oils of host plant after interaction with microbes. We have demonstrated that metabolites and fatty acid content were altered in peanut after interaction with *S*. *maltophilia* BJ01 strain under nitrogen starvation condition. The data presented herein highlight the understanding of different aspects of connection between induced systemic resistance ISR, signaling and metabolic pathways which can play a major role in plant-microbe interaction. Interaction of plants with PGPR’s improve the plant health and soil fertility in many aspects but the systemic information about host plant at metabolic and genetic level is still in infancy which needs an extensive research.

## Supporting information

S1 Fig PCR amplification of *nifH* gene. (TIF)

S2 Fig Plant growth characteristics of peanut plants grown under nitrogen supplement or nitrogen deficit conditions with bacteria inoculum or without inoculum. Estimation of (A) shoot length, (B) root length, (C) fresh weight, and (D) dry weight at different days of the treatment. Bars represent means ± SE.
(TIF)

S3 Fig **Estimation of (A) total antioxidant, (B) DPPH, (C) H**_**2**_**O**_**2**_**, and (D) OH**^**−**^**scavenging activities.** Different activities were measured from peanut plants grown under nitrogen supplement or nitrogen deficit conditions with bacteria inoculum or without inoculum. Bars represent means ± SE.
(TIF)

## References

[1] Singh RP, Jha P, Jha PN. Bio-inoculation of plant growth-promoting *rhizobacterium Enterobacter cloacae* ZNP-3 increased resistance against salt and temperature stresses in wheat plant (Triticum aestivum L.). J Plant Growth Regul. 2017; 36(3):783–98.

[2] Patel BB, Patel BB, Dave RS. Studies on infiltration of saline-alkali soils of several parts of Mehsana and Patan districts of North Gujarat. J Appl Technol Environ Sanit. 2011; 1(1):87–92.

[3] Munns R, James RA. Screening methods for salinity tolerance: a case study with tetraploid wheat. Plant Soil. 2003; 253(1):201–18.

[4] Tester M, Davenport R. Na^+^ tolerance and Na^+^ transport in higher plants. Ann Bot. 2003; 91(5):503–27. 10.1093/aob/mcg058 12646496 PMC4242248

[5] Blaylock AD. Soil salinity, salt tolerance, and growth potential of horticultural and landscape plants. University of Wyoming, Cooperative Extension Service, Department of Plant, Soil, and Insect Sciences, College of Agriculture; 1994.

[6] Mishra A, Patel MK, Jha B. Non-targeted metabolomics and scavenging activity of reactive oxygen species reveal the potential of *Salicornia brachiata* as a functional food. J Funct Food. 2015; 13:21–31.

[7] Patel MK, Mishra A, Jha B. 2016 Untargeted metabolomics of halophytes in *Marine Omics: Principles and Applications*, ed S. Kim (Boca Raton, FL: CRC Press), 309–325. 10.1201/9781315372303-18

[8] Mishra A, Joshi M, Jha B. Oligosaccharide mass profiling of nutritionally important *Salicornia brachiata*, an extreme halophyte. Carbohyd Polym. 2013; 92(2):1942–5. 10.1016/j.carbpol.2012.11.055 23399241

[9] Jha B, Singh NP, Mishra A. Proteome profiling of seed storage proteins reveals the nutritional potential of *Salicornia brachiata* Roxb., an extreme halophyte. J Agr Food Chem. 2012; 60(17):4320–6. 22494338 10.1021/jf203632v

[10] Chaturvedi AK, Mishra A, Tiwari V, Jha B. Cloning and transcript analysis of type 2 metallothionein gene (*SbMT-2*) from extreme halophyte *Salicornia brachiata* and its heterologous expression in *E. coli*. Gene. 2012; 499(2):280–7. 10.1016/j.gene.2012.03.001 22441126

[11] Chaturvedi AK, Patel MK, Mishra A, Tiwari V, Jha B. The SbMT-2 gene from a halophyte confers abiotic stress tolerance and modulates ROS scavenging in transgenic tobacco. PloS one. 2014; 9 (10):e111379 10.1371/journal.pone.0111379 25340650 PMC4207811

[12] Singh N, Mishra A, Jha B. Over-expression of the peroxisomal ascorbate peroxidase (*SbpAPX*) gene cloned from halophyte *Salicornia brachiata* confers salt and drought stress tolerance in transgenic tobacco. Mar Biotechnol. 2014; 16(3):321–32. 10.1007/s10126-013-9548-6 24197564

[13] Singh VK, Mishra A, Haque I, Jha B. A novel transcription factor-like gene *SbSDR1* acts as a molecular switch and confers salt and osmotic endurance to transgenic tobacco. Sci Rep. 2016; 6:31686 10.1038/srep31686 27550641 PMC4994045

[14] Patel MK, Joshi M, Mishra A, Jha B. Ectopic expression of *SbNHX1* gene in transgenic castor (*Ricinus communis* L.) enhances salt stress by modulating physiological process. Plant Cell Tiss Org. 2015; 122(2):477–90.

[15] Pandey S, Patel MK, Mishra A, Jha B. In planta transformed cumin (*Cuminum cyminum* L.) plants, overexpressing the *SbNHX1* gene showed enhanced salt endurance. PloS one. 2016; 11(7):e0159349 10.1371/journal.pone.0159349 27411057 PMC4943630

[16] Udawat P, Mishra A, Jha B. Heterologous expression of an uncharacterized universal stress protein gene (*SbUSP*) from the extreme halophyte, *Salicornia brachiata*, which confers salt and osmotic tolerance to E. *coli*. Gene. 2014; 536(1):163–70. 10.1016/j.gene.2013.11.020 24291028

[17] Udawat P, Jha RK, Sinha D, Mishra A, Jha B. Overexpression of a cytosolic abiotic stress responsive universal stress protein (SbUSP) mitigates salt and osmotic stress in transgenic tobacco plants. Front Plant Sci. 2016; 7:518 10.3389/fpls.2016.00518 27148338 PMC4838607

[18] Udawat P, Jha RK, Mishra A, Jha B. Overexpression of a plasma membrane-localized SbSRP-like protein enhances salinity and osmotic stress tolerance in transgenic tobacco. Front Plant Sci. 2017; 8:582 10.3389/fpls.2017.00582 28473839 PMC5397517

[19] Mishra A, Tanna B. Halophytes: potential resources for salt stress tolerance genes and promoters. Front Plant Sci. 2017; 8:829 10.3389/fpls.2017.00829 28572812 PMC5435751

[20] Jha B, Mishra A, Jha A, Joshi M. Developing transgenic Jatropha using the *SbNHX1* gene from an extreme halophyte for cultivation in saline wasteland. PLoS One. 2013; 8(8):e71136 10.1371/journal.pone.0071136 23940703 PMC3733712

[21] Jha B, Sharma A, Mishra A. Expression of *SbGSTU* (tau class glutathione S-transferase) gene isolated from *Salicornia brachiata* in tobacco for salt tolerance. Mol Biol Rep. 2011; 38(7):4823–32. 10.1007/s11033-010-0625-x 21136169

[22] Jha RK, Patel J, Mishra A, Jha B. 2019 Introgression of halophytic salt stress-responsive genes for developing stress tolerance in crop plants In: Hasanuzzaman M, Shabala S, and Fujita M (Eds.*)* *Halophytes and climate change*: *adaptive mechanisms and potential uses*, CABI, UK, pp. 288–299. 10.1079/9781786394330.0275

[23] Tiwari V, Chaturvedi AK, Mishra A, Jha B. The transcriptional regulatory mechanism of the peroxisomal ascorbate peroxidase (*pAPX*) gene cloned from an extreme halophyte, *Salicornia brachiata*. Plant Cell Physiol. 2013; 55(1):201–17. 10.1093/pcp/pct172 24285755

[24] Tiwari V, Patel MK, Chaturvedi AK, Mishra A, Jha B. Functional characterization of the tau class glutathione-S-transferases gene (*SbGSTU*) promoter of *Salicornia brachiata* under salinity and osmotic stress. PLoS One. 2016; 11(2):e0148494 10.1371/journal.pone.0148494 26885663 PMC4757536

[25] Tiwari V, Patel MK, Chaturvedi AK, Mishra A, Jha B. Cloning and functional characterization of the Na^+^/H^+^ antiporter (*NHX1*) gene promoter from an extreme halophyte *Salicornia brachiata*. Gene. 2019; 683:233–42. 10.1016/j.gene.2018.10.039 30340051

[26] Patel MK, Pandey S, Brahmbhatt HR, Mishra A, Jha B. Lipid content and fatty acid profile of selected halophytic plants reveal a promising source of renewable energy. Biomass and Bioenergy. 2019; 124, 25–32.

[27] Alexander A, Mishra A, Jha B. 2019 Halotolerant Rhizobacteria: A Promising Probiotic for Saline Soil-Based Agriculture In: Kumar M., Etesami H., Kumar V. (eds) *Saline Soil-based Agriculture by Halotolerant Microorganisms*. Springer, Singapore, pp. 53–73. 10.1007/978-981-13-8335-9_3

[28] Singh VK, Kavita K, Prabhakaran R, Jha B. Cis-9-octadecenoic acid from the rhizospheric bacterium *Stenotrophomonas maltophilia* BJ01 shows quorum quenching and anti-biofilm activities. Biofouling. 2013; 29(7):855–67. 10.1080/08927014.2013.807914 23844805

[29] Ryan RP, Monchy S, Cardinale M, Taghavi S, Crossman L, Avison MB, Berg G, Van Der Lelie D, Dow JM. The versatility and adaptation of bacteria from the genus *Stenotrophomonas*. Nat Rev Microbiol. 2009; 7(7):514 10.1038/nrmicro2163 19528958

[30] Singh VK, Mishra A, Jha B. Anti-quorum sensing and anti-biofilm activity of *Delftia tsuruhatensis* extract by attenuating the quorum sensing-controlled virulence factor production in *Pseudomonas aeruginosa*. Front Cell Infect Microbiol. 2017; 7:337 10.3389/fcimb.2017.00337 28798903 PMC5526841

[31] Singh VK, Mishra A, Jha B. 3-Benzyl-hexahydro-pyrrolo [1, 2-a] pyrazine-1, 4-dione extracted from *Exiguobacterium indicum* showed anti-biofilm activity against Pseudomonas aeruginosa by attenuating quorum sensing. Frontiers in Microbiology, 2019; 10:1269 10.3389/fmicb.2019.01269 31231348 PMC6568026

[32] Singh RP, Jha P N. The PGPR *Stenotrophomonas maltophilia* SBP-9 augments resistance against biotic and abiotic stress in wheat plants. Frontiers in Microbiology, 2017, 8, 1945 10.3389/fmicb.2017.01945 29062306 PMC5640710

[33] Pandey MK, Monyo E, Ozias-Akins P, Liang X, Guimarães P, Nigam SN, Upadhyaya HD, Janila P, Zhang X, Guo B, Cook DR. Advances in Arachis genomics for peanut improvement. Biotechnol Adv. 2012; 30(3):639–51. 10.1016/j.biotechadv.2011.11.001 22094114

[34] Singh N, Mishra A, Jha B. Ectopic over-expression of peroxisomal ascorbate peroxidase (*SbpAPX*) gene confers salt stress tolerance in transgenic peanut (*Arachis hypogaea*). Gene. 2014; 547(1):119–25. 10.1016/j.gene.2014.06.037 24954532

[35] Tiwari V, Chaturvedi AK, Mishra A, Jha B. An efficient method of Agrobacterium-mediated genetic transformation and regeneration in local Indian cultivar of groundnut (Arachis hypogaea) using grafting. Appl Biochem Biotech. 2015; 175(1):436–53. 10.1007/s12010-014-1286-3 25308617

[36] Tiwari V, Chaturvedi AK, Mishra A, Jha B. Introgression of the *SbASR-1* gene cloned from a halophyte *Salicornia brachiata* enhances salinity and drought endurance in transgenic groundnut (*Arachis hypogaea*) and acts as a transcription factor. PLoS One. 2015; 10(7):e0131567 10.1371/journal.pone.0131567 26158616 PMC4497679

[37] Jha B, Mishra A, Chaturvedi AK. Engineering Stress Tolerance in Peanut (*Arachis hypogaea* L.) In: Watson R, Preedy VR(Eds.) Genetically Modified Organisms (GMO) Foods: Production, Regulation and Public Health 2016 (pp. 305–311). Elsevier, Philadelphia, USA.

[38] Yousuf B, Keshri J, Mishra A, Jha B. Application of targeted metagenomics to explore abundance and diversity of CO2-fixing bacterial community using *cbbL* gene from the rhizosphere of *Arachis hypogaea*. Gene. 2012; 506(1):18–24. 10.1016/j.gene.2012.06.083 22766402

[39] Keshri J, Mishra A, Jha B. Microbial population index and community structure in saline–alkaline soil using gene targeted metagenomics. Microbiological Research, 2013; 168(3):165–173. 10.1016/j.micres.2012.09.005 23083746

[40] Arnon DI. Copper enzymes in isolated chloroplasts. Polyphenoloxidase in *Beta vulgaris*. Plant physiol. 1949; 24(1):1 16654194 10.1104/pp.24.1.1 PMC437905

[41] Chamovitz D, Sandmann G, Hirschberg J. Molecular and biochemical characterization of herbicide-resistant mutants of cyanobacteria reveals that phytoene desaturation is a rate-limiting step in carotenoid biosynthesis. J Biol Chem. 1993; 268(23):17348–53. 8349618

[42] Lutts S, Kinet JM, Bouharmont J. NaCl-induced senescence in leaves of rice (*Oryza sativa* L.) cultivars differing in salinity resistance. Ann Bot. 1996; 78(3):389–98.

[43] Hayat S, Yadav S, Ali B, Ahmad A. Interactive effect of nitric oxide and brassinosteroids on photosynthesis and the antioxidant system of *Lycopersicon esculentum*. Russ J Plant Physl. 2010; 57(2):212–21.

[44] Hodges DM, DeLong JM, Forney CF, Prange RK. Improving the thiobarbituric acid-reactive-substances assay for estimating lipid peroxidation in plant tissues containing anthocyanin and other interfering compounds. Planta. 1999; 207(4):604–11. 10.1007/s00425-017-2699-3 28456836

[45] Mukherjee SP, Choudhuri MA. Implications of water stress‐induced changes in the levels of endogenous ascorbic acid and hydrogen peroxide in Vigna seedlings. Physiol Plantarum. 1983; 58(2):166–70.

[46] Andreae WA, Van Ysselstein MW. Studies on 3-indoleacetic acid metabolism. V. Effect of calcium ions on 3-indoleacetic acid uptake and metabolism by pea roots. Plant physiol. 1960; 35(2):220 16655332 10.1104/pp.35.2.220 PMC405946

[47] Bates LS, Waldren RP, Teare ID. Rapid determination of free proline for water-stress studies. Plant soil. 1973; 39(1):205–7.

[48] Patel MK, Mishra A, Jha B. Non-targeted metabolite profiling and scavenging activity unveil the nutraceutical potential of psyllium (*Plantago ovata* Forsk). Frontiers in Plant Science. 2016; 7:431 10.3389/fpls.2016.00431 27092153 PMC4821064

[49] Pandey S, Patel MK, Mishra A, Jha B. Physio-biochemical composition and untargeted metabolomics of cumin (*Cuminum cyminum* L.) make it promising functional food and help in mitigating salinity stress. PLoS One, 2015; 10(12):e0144469 10.1371/journal.pone.0144469 26641494 PMC4671573

[50] Hazra B, Biswas S, Mandal N. Antioxidant and free radical scavenging activity of *Spondias pinnata*. BMC Complem Altern M. 2008; 8(1):63. 10.1186/1472-6882-8-63 PMC2636748 19068130

[51] Tanna B, Choudhary B, Mishra A. Metabolite profiling, antioxidant, scavenging and anti-proliferative activities of selected tropical green seaweeds reveal the nutraceutical potential of *Caulerpa* spp. Algal Res. 2018; 36:96–105.

[52] Zhishen J, Mengcheng T, Jianming W. The determination of flavonoid contents in mulberry and their scavenging effects on superoxide radicals. Food Chem. 1999; 64(4):555–9.

[53] Re R, Pellegrini N, Proteggente A, Pannala A, Yang M, Rice-Evans C. Antioxidant activity applying an improved ABTS radical cation decolorization assay. Free radical Bio Med. 1999; 26(9–10):1231–7. 10381194 10.1016/s0891-5849(98)00315-3

[54] Saeed N, Khan MR, Shabbir M. Antioxidant activity, total phenolic and total flavonoid contents of whole plant extracts *Torilis leptophylla* L. BMC Complem Altern M. 2012; 12(1):221. 10.1186/1472-6882-12-221 PMC3524761 23153304

[55] Yousuf B, Kumar R, Mishra A, Jha B. Differential distribution and abundance of diazotrophic bacterial communities across different soil niches using a gene-targeted clone library approach. FEMS Microbiol Lett. 2014; 360(2):117–25. 10.1111/1574-6968.12593 25196726

[56] Keshri J, Yousuf B, Mishra A, Jha B. The abundance of functional genes, *cbbL*, *nifH*, *amoA* and *apsA*, and bacterial community structure of intertidal soil from Arabian Sea. Microbiol Res. 2015; 175:57–66. 10.1016/j.micres.2015.02.007 25862282

[57] Kanehara S, Ohtani T, Uede K, Furukawa F. Clinical effects of undershirts coated with borage oil on children with atopic dermatitis: A double‐blind, placebo‐controlled clinical trial. The J Dermatol. 2007; 34(12):811–5. 18078406 10.1111/j.1346-8138.2007.00391.x

[58] Dubois V, Breton S, Linder M, Fanni J, Parmentier M. Fatty acid profiles of 80 vegetable oils with regard to their nutritional potential. Eur J Lipid Sci Tech. 2007; 109(7):710–32.

[59] Zheng CJ, Yoo JS, Lee TG, Cho HY, Kim YH, Kim WG. Fatty acid synthesis is a target for antibacterial activity of unsaturated fatty acids. FEBS Lett. 2005; 579(23):5157–62. 16146629 10.1016/j.febslet.2005.08.028

[60] Silva LR, Pereira MJ, Azevedo J, Mulas R, Velazquez E, González-Andrés F, Valentão P, Andrade PB. Inoculation with *Bradyrhizobium japonicum* enhances the organic and fatty acids content of soybean (*Glycine max* (L.) Merrill) seeds. Food Chem. 2013; 141(4):3636–48. 10.1016/j.foodchem.2013.06.045 23993531

[61] Brechenmacher L, Lei Z, Libault M, Findley S, Sugawara M, Sadowsky MJ, Sumner LW, Stacey G. Soybean metabolites regulated in root hairs in response to the symbiotic bacterium *Bradyrhizobium japonicum*. Plant Physiol. 2010; 153(4):1808–22. 10.1104/pp.110.157800 20534735 PMC2923908

[62] Cagide C, Riviezzi B, Minteguiaga M, Morel MA, Castro-Sowinski S. Identification of Plant Compounds Involved in the Microbe-Plant Communication During the Coinoculation of Soybean with *Bradyrhizobium elkanii* and *Delftia* sp. strain JD2. Mol Plant Microbe In. 2018; 31(11):1192–9. 10.1094/MPMI-04-18-0080-CR 29845886

[63] Morel MA, Cagide C, Minteguiaga MA, Dardanelli MS, Castro-Sowinski S. The pattern of secreted molecules during the co-inoculation of alfalfa plants with Sinorhizobium meliloti and Delftia sp. strain JD2: an interaction that improves plant yield. Mol Plant Microbe In. 2015; 28(2):134–42. 10.1094/MPMI-08-14-0229-R 25353366

[64] Morel MA, Braña V, Castro-Sowinski S. Legume crops, importance and use of bacterial inoculation to increase production. InCrop plant 2012. IntechOpen.

[65] Ripullone F, Grassi G, Lauteri M, Borghetti M. Photosynthesis–nitrogen relationships: interpretation of different patterns between *Pseudotsuga menziesii* and *Populus euroamericana* in a mini-stand experiment. Tree Physiol. 2003; 23(2):137–44. 12533308 10.1093/treephys/23.2.137

[66] Nisar N, Li L, Lu S, Khin NC, Pogson BJ. Carotenoid metabolism in plants. Mol Plant. 2015; 8(1):68–82. 10.1016/j.molp.2014.12.007 25578273

[67] Bashan Y, Bustillos JJ, Leyva LA, Hernandez JP, Bacilio M. Increase in auxiliary photoprotective photosynthetic pigments in wheat seedlings induced by *Azospirillum brasilense*. Biol Fert Soils. 2006; 42(4):279–85.

[68] Cohen AC, Bottini R, Pontin M, Berli FJ, Moreno D, Boccanlandro H, Travaglia CN, Piccoli PN. *Azospirillum brasilense* ameliorates the response of *Arabidopsis thaliana* to drought mainly via enhancement of ABA levels. Physiol Plantarum. 2015; 153(1):79–90. 10.1111/ppl.12221 24796562

[69] Claussen W. Proline as a measure of stress in tomato plants. Plant Sci. 2005; 168(1):241–8.

[70] Koca H, Ozdemir F, Turkan I. Effect of salt stress on lipid peroxidation and superoxide dismutase and peroxidase activities of *Lycopersicon esculentum* and *L. pennellii*. Biol Plantarum. 2006; 50(4):745–8.

[71] Yazici I, Türkan I, Sekmen AH, Demiral T. Salinity tolerance of purslane (*Portulaca oleracea* L.) is achieved by enhanced antioxidative system, lower level of lipid peroxidation and proline accumulation. Environ Exp Bot. 2007; 61(1):49–57.

[72] Tanna B, Mishra A. Metabolites unravel nutraceutical potential of edible seaweeds: an emerging source of functional food. Comprehensive Reviews in Food Science and Food Safety, 2018; 17(6):1613–1624. 10.1111/1541-4337.12396 33350143

[73] Seneviratne G, Jayasinghearachchi HS. Phenolic acids: Possible agents of modifying N 2-fixing symbiosis through rhizobial alteration?. Plant soil. 2003; 252(2):385–95.

[74] Makoi JH, Ndakidemi PA. Biological, ecological and agronomic significance of plant phenolic compounds in rhizosphere of the symbiotic legumes. Afr J Biotechnol. 2007; 6(12).

[75] Juge C, Prévost D, Bertrand A, Bipfubusa M, Chalifour FP. Growth and biochemical responses of soybean to double and triple microbial associations with *Bradyrhizobium, Azospirillum* and arbuscular mycorrhizae. Appl Soil Ecol. 2012; 61:147–57.

[76] Antunes PM, Rajcan I, Goss MJ. Specific flavonoids as interconnecting signals in the tripartite symbiosis formed by arbuscular mycorrhizal fungi, *Bradyrhizobium japonicum* (Kirchner) Jordan and soybean (*Glycine max* (L.) Merr.). Soil Biol Biochem. 2006; 38(3):533–43.

[77] Chang C, Damiani I, Puppo A, Frendo P. Redox changes during the legume–Rhizobium symbiosis. Mol Plant. 2009; 2(3):370–7. 10.1093/mp/ssn090 19825622

[78] Prakash D, Singh BN, Upadhyay G. Antioxidant and free radical scavenging activities of phenols from onion (*Allium cepa*). Food chem. 2007; 102(4):1389–93.

[79] Kim JA, Jung WS, Chun SC, Yu CY, Ma KH, Gwag JG, Chung IM. A correlation between the level of phenolic compounds and the antioxidant capacity in cooked-with-rice and vegetable soybean (*Glycine max* L.) varieties. Eur Food Res Technol. 2006; 224(2):259–70.

[80] Sakthivelu G, Akitha Devi MK, Giridhar P, Rajasekaran T, Ravishankar GA, Nikolova MT, Angelov GB, Todorova RM, Kosturkova GP. Isoflavone composition, phenol content, and antioxidant activity of soybean seeds from India and Bulgaria. J Agr Food Chem. 2008; 56(6):2090–5. 18303813 10.1021/jf072939a

