# Supplementary figures and images for: Plant growth promoting rhizobacterium Stenotrophomonas maltophilia BJ01 augments endurance against N2 starvation by modulating physiology and biochemical activities of Arachis hypogea
Source: PLoS One. 2019 Sep 12;14(9):e0222405. doi: 10.1371/journal.pone.0222405 (PMC6742461; doi:10.1371/journal.pone.0222405)

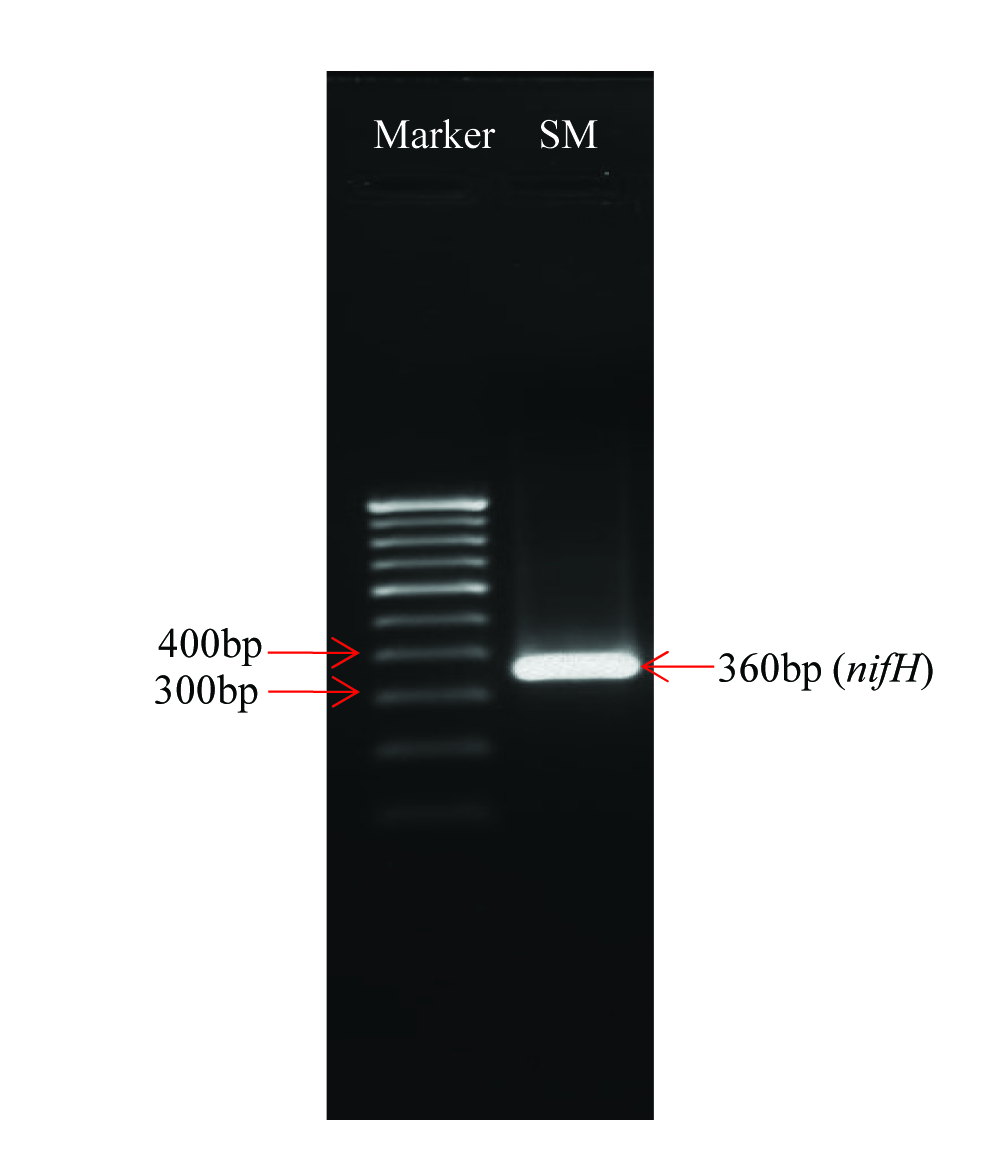

Supplement: S1 Fig — (TIF) [file pone.0222405.s001.tif]

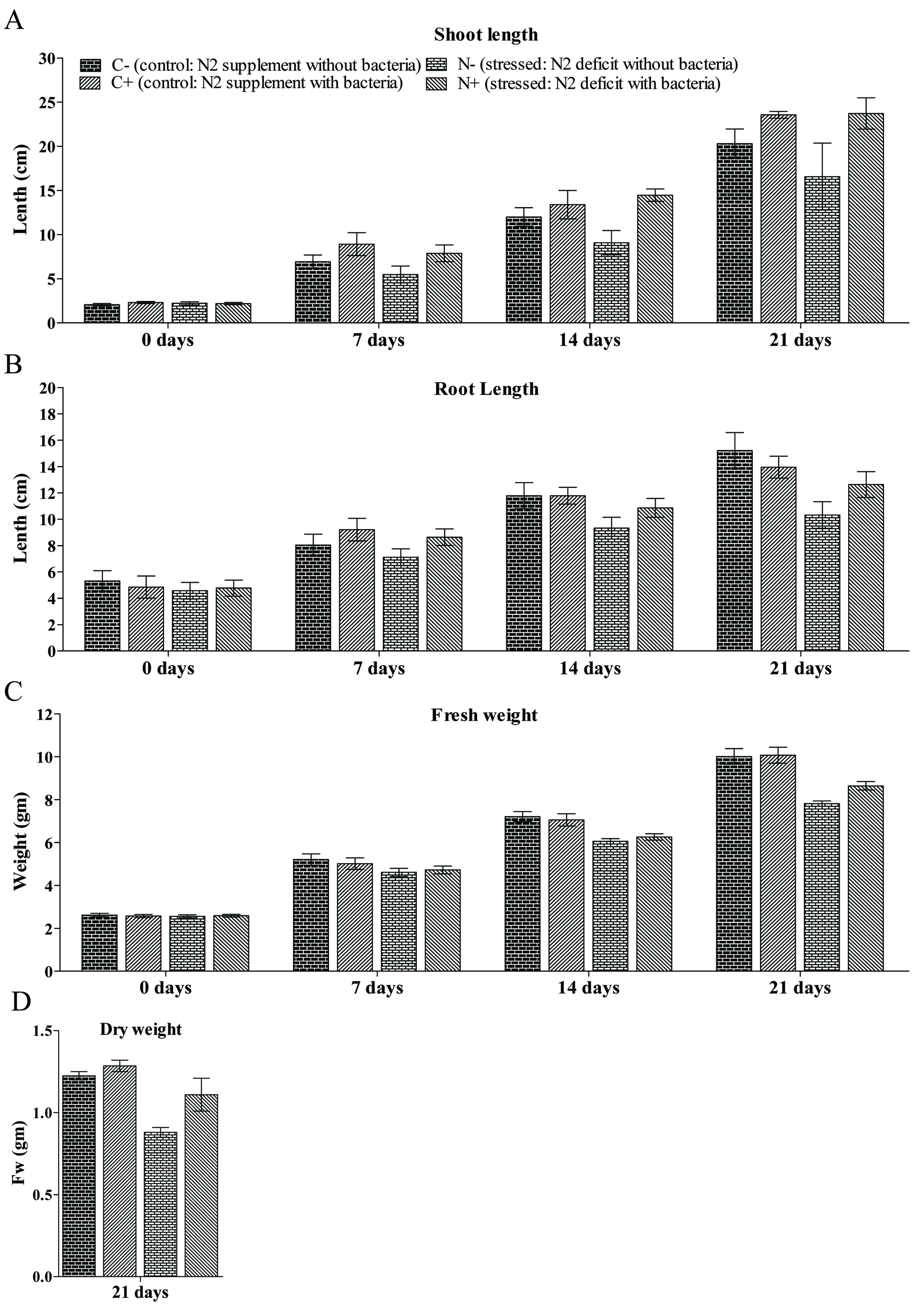

Supplement: S2 Fig — Estimation of (A) shoot length, (B) root length, (C) fresh weight, and (D) dry weight at different days of the treatment. Bars represent means ± SE. (TIF) [file pone.0222405.s002.tif]

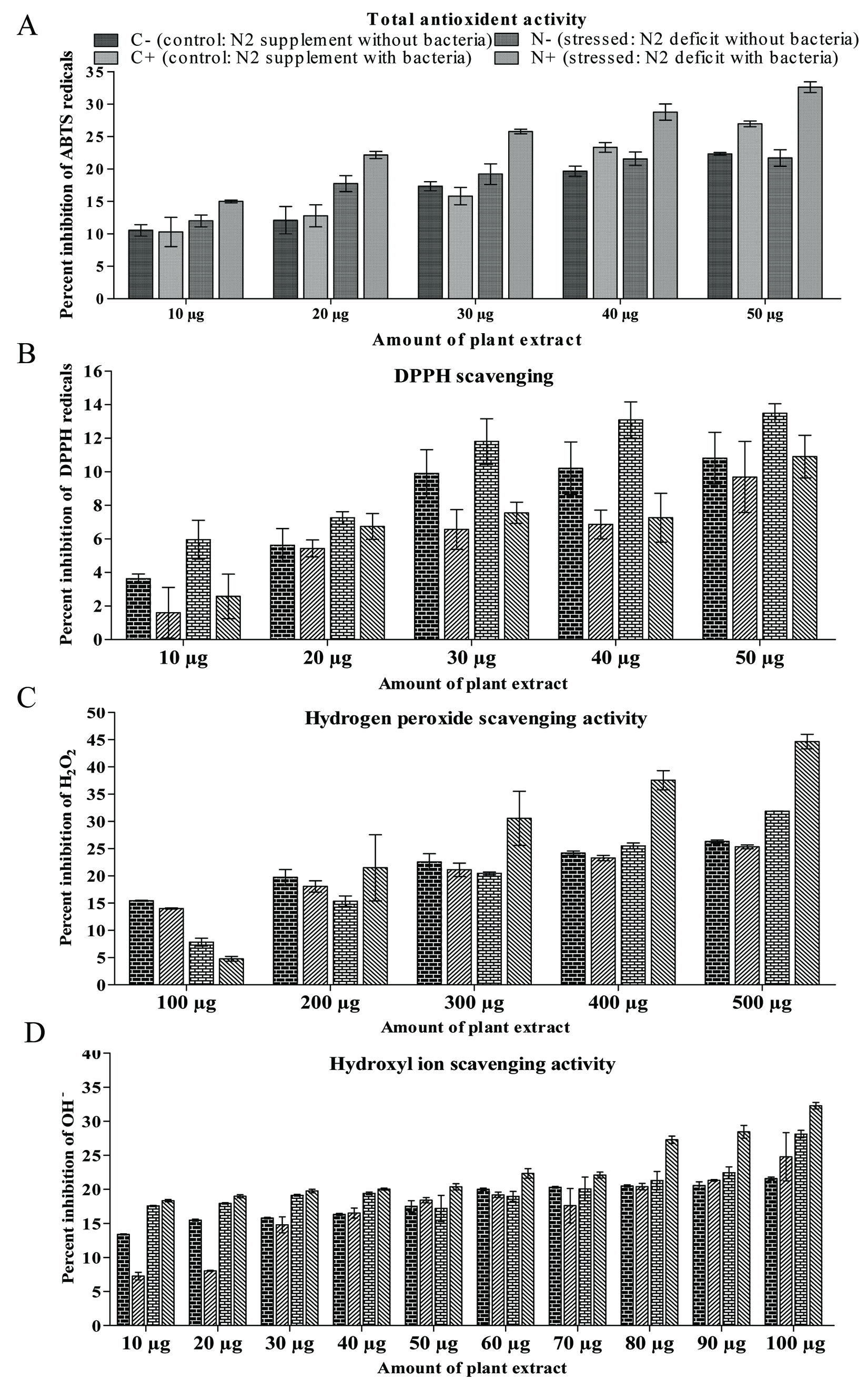

Supplement: S3 Fig — Estimation of (A) total antioxidant, (B) DPPH, (C) H2O2, and (D) OH−scavenging activities. Different activities were measured from peanut plants grown under nitrogen supplement or nitrogen deficit conditions with bacteria inoculum or without inoculum. Bars represent means ± SE. (TIF) [file pone.0222405.s003.tif]
